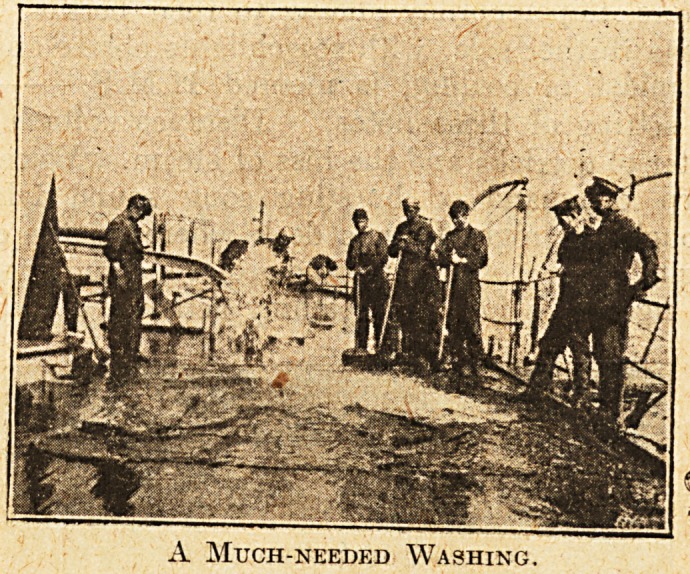# A Ship's Dark Hours: Coaling

**Published:** 1919-03-22

**Authors:** 


### NOTES OF A NAVAL SURGEON.

### I.—A Ship's Dark Hours: Coaling.

THE width of the basin lies before you; black, glittering water bearing the shapes of even blacker drifters, imperceptibly gliding to their berths, and on the further side, with searchlights ablaze and in the midst of coaling, is moored H.M.S. ———, the setting of these notes. Distance softens the clatter of derricks and the hiss of steam. Voices in sharp command or bursting song sound faintly through the night from a deck where, in the converging rays, work a thousand grotesque and gnome-like figures, a focus of seething activity, a million

A MIDNIGHT COALING.

times intensified by the dazzling light and a frame-velvet darkness. Watching the flickering clouds of dust and vapour momentarily caught in brilliance, the reflections and the searchlight beams as they escape, show for an instant some vague corner of the yard, some unsuspected ship or caisson and then spring back to that thronging deck, the mind is impressed by a marvellous and arresting beauty.

But once across the gangway, the awakening is abrupt. Coal, coal, and yet more coal; coal-dust inches deep on deck, in every breath, in every crevice! Trolleys, bags, shovels, clanging machinery and perspiring, blackened men in costumes, originally "fancy"—a time honoured matelot's joke—but now indistinguishable from their wearers' inky skins, make turmoil indescribable and appalling.

Regulations wisely require that a surgeon shall be aboard at these times when circumstance brings an inevitable train of minor accidents, and it is very noticeable that after long hours of such labour, even when watches work in turn, exhaustion has a strong tendency to increase the incidence of casualties. Exposure on the upper-deck for so long a time in bad weather too frequently causes collapse among the weaker men and a careful eye must be kept on any showing cardiac or bronchitic deficiencies. On one well-remembered day the thermometer was registering minus 10 degrees Fahr. and the cold was accentuated by a piercing wind, from which the men had little shelter. Overnight ice-breakers were called to get the coal-lighter alongside and by morning both these and the ship found themselves firmly frozen in. Two men falling from the ship's side struck, instead of the "ditch," ice several inches thick, and many were the cases of frostbite through standing for hours in exposed control positions. In the days following sick-bay was relatively inundated by the after-effects of that one day's labour.

A common cause of injury while coaling is the breaking of hoists under strain. The force in a flying wire-end is sometimes amazing. A complicated series is rigged to support a line of pulleys for lifting batches of a dozen or more loaded bags from the depths of the lighter and besides the armies continually at work with shovel and trolley, many officers and senior ratings are stationed at points of vantage to superintend operations. One has seen a man struck full on the chest and brought to the deck twenty feet below, suffering fractures of several ribs and the right lower leg bones, while on another occasion, missing an officer by inches, the metal smashed the supporting gear of an adjacent searchlight. A method with the quality of increasing risk as well as speed consists of tipping complete rail-loads of coal on to the ship. The truck is hoisted bodily in-board, an apparent avalanche of coal falls crashing on the deck and a watchful eye indeed must be kept on those bounding fragments.

In H.M.S.———, the sick-bay is crowded away forrard under the fo'-castle. At best an inaccessible spot, but with all except two hatches closed to keep out the showering dust, it is even more difficult than usual to reach in a coaling emergency. By

WIRES AND DERRICKS.

the time any case arrives in this haven, a wound is literally plastered with fine coal powder, but, strange to say, sepsis is not a common occurrence. Man-handling is an indescribably grimy process, and it is a truly extraordinary sight to watch a first-aid administration by a negroid group whose teeth and sclerotics form the only break in a total blackness. This little hospital presents a sorry sight after one of these invasions.

The most primitive and decidedly the most

**Notes of a Naval Surgeon**—(*continued*).

laborious way in which one has ever seen the sailor called upon to get his coal aboard was by means of small baskets carried shoulder high. The ship alongside was even then some hundred yards away from her appointed twelve hundred tons with a stretch of wharf intervening, flat, but littered with sundry ships' fittings and huge spare parts. At daybreak the procession commenced; rows of figures moving like two endless chains to and from the ship. Brief spells of rest, a well-meaning ship's band perched on the top of a disused boiler, and the starting of every popular song of the day may have done something to relieve the strain and monotony, and fair weather and absence of machinery kept the casualty list at a minimum, but it was far into the night that seven hundred dog-tired men found their day's work over. The reader will picture the coaling duties of most of the officers as orders and organisation. To some extent he is correct, but there is perhaps no time apart from actual fighting at which the "lower deck" are more helped by human understanding and personal example from their seniors. Padre will shovel and pull with the men in the dungeon-like hold of a lighter, and truly it may be

An Avalanche of Coal.

said that no one asks of a man what he is unwilling to do himself.

Probably the greatest physical strain falls on the stokers below in the bunkers, working amidst heat and an atmosphere indescribable. Their only exit is a small round manhole, at their side falls a stream of coal shot down from above. Forced ventilation is so vital that a break-down may be followed by the most disastrous results. One remembers an instance where, owing to a gradual list of the ship, a store of sacked potatoes shifted at a neighbouring bulk-head, and obstructed the air-way to the after bunker. Within a few minutes the men were overcome and the

A Much-needed Washing.

chief stoker and a P.O., attempting rescue, met with a like fate. Getting the inlet clear and hoisting the unconscious men through a two-and-a-half foot opening filled some exciting moments and it was with considerable relief that one saw the last of them open his eyes after strychnine, oxygen, and much artificial respiration. Of all ship's duties, stoking work is undoubtedly the most exacting, and many are the cardiac cases which first come to knowledge through this department. Probably long custom breeds indifference, but nevertheless, it was with lasting admiration that the author watched the labours of these men, and the lightheartedness with which they carried on under conditions of seeming impossible severity. Near ice-bound shores or in the tropics, the same routine, the same long hours of shovelling, hoisting and pushing with aid from not one of the labour-saving devices of the modern liner. The warship is adapted to the simplest mechanical means. Complicated machinery may break down and therefore she, with her relatively larger crew, relies on bags, wires, pulleys, and strong arms. Sometimes a collier helps her and she is grateful, but the warship can always fend for herself and all honour to stout men who give her that power.

## Figures and Tables

**Figure f1:**
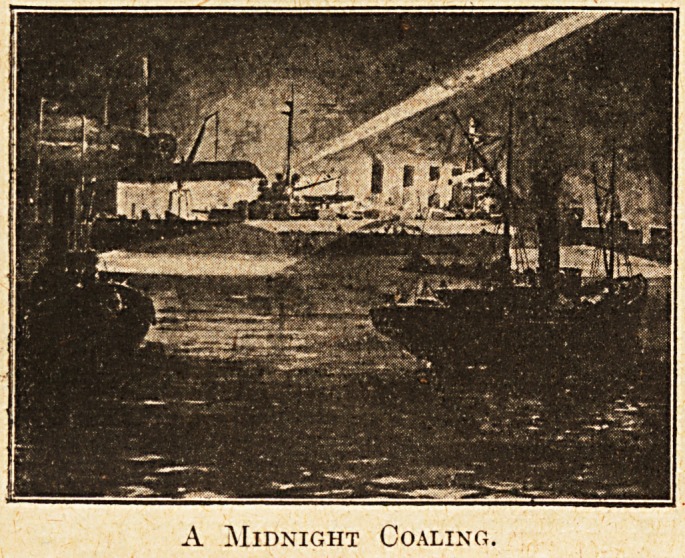


**Figure f2:**
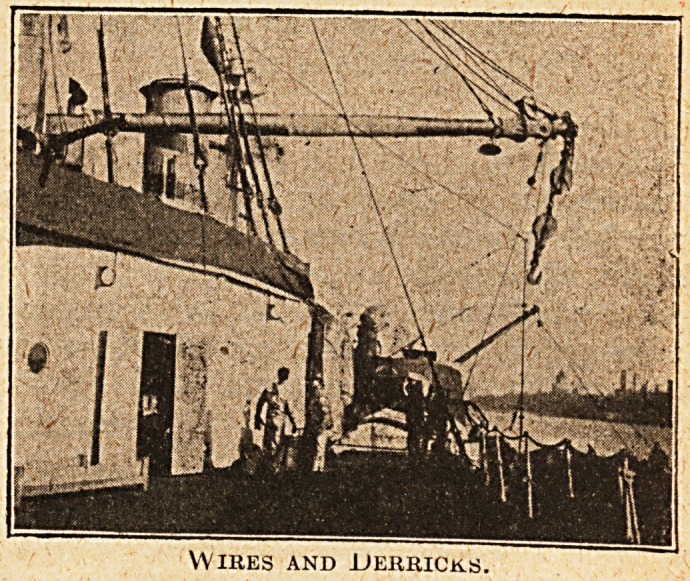


**Figure f3:**
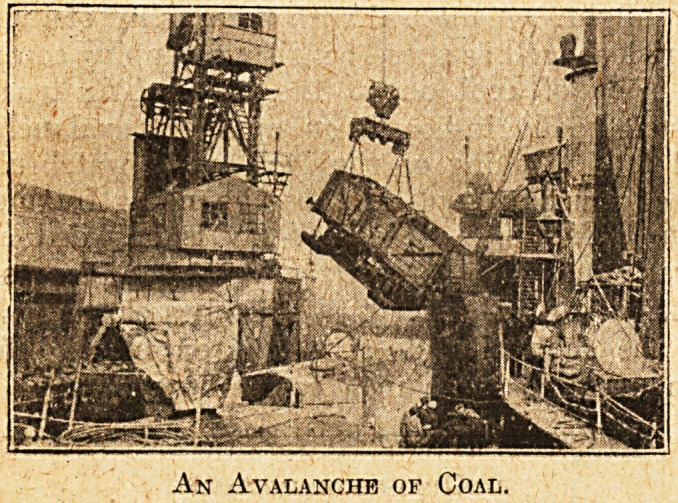


**Figure f4:**